# Tri­phenyl­tellurium chloride

**DOI:** 10.1107/S160053681400498X

**Published:** 2014-03-12

**Authors:** Ambika Chopra, Shalini Jain, Sanjay K. Srivastava, Sushil K. Gupta, Ray J. Butcher

**Affiliations:** a School of Studies in Chemistry, Jiwaji University, Gwalior 474011, India; b Department of Chemistry, Howard University, 525 College Street NW, Washington, DC 20059, USA

## Abstract

The asymmetric unit of the title compound, C_18_H_15_ClTe, contains two mol­ecules which are in inverted orientations. The compound displays a tetra­hedral geometry around the Te atom in spite of there being five electron domains. This is attributed to the fact that the lone pair is not sterically active. The dihedral angles between the three phenyl rings are 76.51 (16)/73.75 (16)/71.06 (17) and 78.60 (17)/77.67 (16)/79.11 (16)° in the two mol­ecules. The crystal packing features eight C—H⋯π inter­actions.

## Related literature   

For the first synthesis of the title compound, see: Günther *et al.* (1974 ▶). For related compounds, see: Klapötke *et al.* (2001 ▶); Naumann *et al.* (2002 ▶). For chalcogen-bearing compounds, see: Srivastava *et al.* (2010 ▶, 2011 ▶); Rastogi *et al.* (2011 ▶). For organotellurium(IV) derivatives that form metal complexes and supra­molecular aggregations, see: Santos *et al.* (2007 ▶); Teikink & Zukerman-Schpector (2010 ▶). For their applications as anti­leishmanial and anti­bacterial agents, see: Lima *et al.* (2009 ▶); Soni *et al.* (2005 ▶).
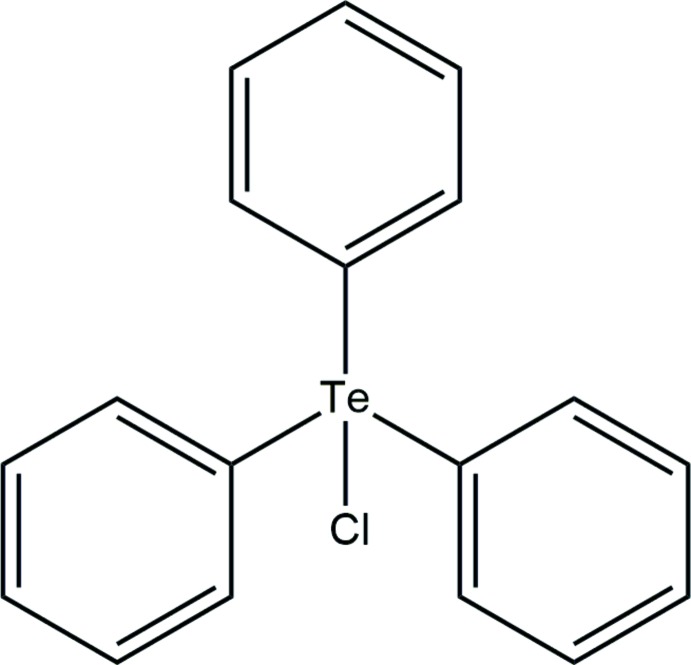


## Experimental   

### 

#### Crystal data  

C_18_H_15_ClTe
*M* *_r_* = 394.35
Monoclinic, 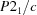

*a* = 18.7514 (3) Å
*b* = 9.60800 (15) Å
*c* = 18.4367 (3) Å
β = 105.2453 (16)°
*V* = 3204.74 (9) Å^3^
*Z* = 8
Cu *K*α radiation
μ = 16.07 mm^−1^
*T* = 123 K
0.25 × 0.12 × 0.08 mm

#### Data collection  

Agilent Xcalibur (Ruby, Gemini) diffractometer
Absorption correction: multi-scan (*CrysAlis PRO*; Agilent, 2012 ▶) *T*_min_ = 0.215, *T*_max_ = 1.000
12435 measured reflections
6446 independent reflections
5854 reflections with *I* > 2σ(*I*)
*R*_int_ = 0.051

#### Refinement  

*R*[*F* ^2^ > 2σ(*F* ^2^)] = 0.034
*wR*(*F* ^2^) = 0.077
*S* = 1.04
6446 reflections
361 parameters
H-atom parameters constrained
Δρ_max_ = 0.98 e Å^−3^
Δρ_min_ = −1.24 e Å^−3^

### 

Data collection: *CrysAlis PRO* (Agilent, 2012 ▶); cell refinement: *CrysAlis PRO*; data reduction: *CrysAlis PRO*; program(s) used to solve structure: *SHELXS2013* (Sheldrick, 2008 ▶); program(s) used to refine structure: *SHELXL2013* (Sheldrick, 2008 ▶); molecular graphics: *SHELXTL* (Sheldrick, 2008 ▶); software used to prepare material for publication: *SHELXTL*.

## Supplementary Material

Crystal structure: contains datablock(s) I. DOI: 10.1107/S160053681400498X/jj2184sup1.cif

Structure factors: contains datablock(s) I. DOI: 10.1107/S160053681400498X/jj2184Isup2.hkl

Supporting information file. DOI: 10.1107/S160053681400498X/jj2184Isup3.cml

CCDC reference: 990042

Additional supporting information:  crystallographic information; 3D view; checkCIF report

## Figures and Tables

**Table 1:** Hydrogen-bond geometry (Å, °) *Cg*1, *Cg*2, *Cg*3, *Cg*4, *Cg*5 and *Cg*6 are the centroids of the C1*A*–C6*A*, C7*A*–C12*A*, C13*A*–C18*A*, C1*B*–C6*B*, C7*B*–C12*B* and C13*B*–C18*B* phenyl rings, respectively.

*D*—H⋯*A*	*D*—H	H⋯*A*	*D*⋯*A*	*D*—H⋯*A*
C2*A*—H2*AA*⋯*Cg*2^i^	0.95	2.96	3.587 (4)	125
C5*A*—H5*AA*⋯*Cg*4	0.95	2.65	3.497 (4)	149
C10*A*—H10*A*⋯*Cg*1^ii^	0.95	2.83	3.580 (4)	137
C5*B*—H5*BA*⋯*Cg*5^iii^	0.95	2.76	3.532 (4)	139
C11*A*—H11*A*⋯*Cg*3^iv^	0.95	2.91	3.601 (4)	131
C12*B*—H12*B*⋯*Cg*6^v^	0.95	2.95	3.671 (3)	134
C14*B*—H14*B*⋯*Cg*4^vi^	0.95	2.86	3.589 (4)	134
C17*B*—H17*B*⋯*Cg*2	0.95	2.78	3.679 (4)	158

Symmetry codes: (i) 
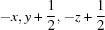
; (ii) 
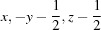
; (iii) 
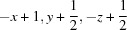
; (iv) 
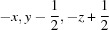
; (v) 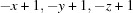; (vi) 
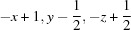
.

**Fig. 1.**
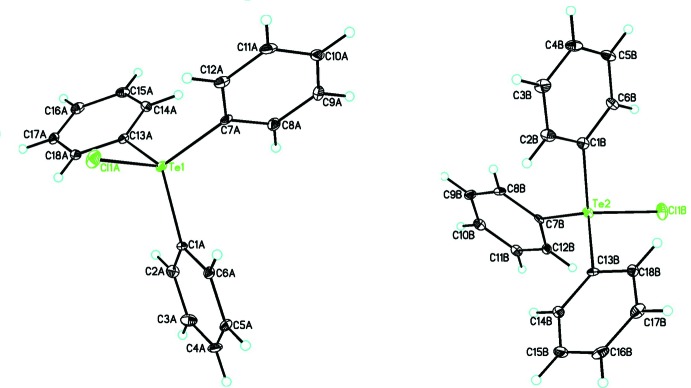
Molecular structure of the title compound showing atom numbering scheme. Displacement ellipsoids are drawn at the 50% probability level.

**Fig. 2.**
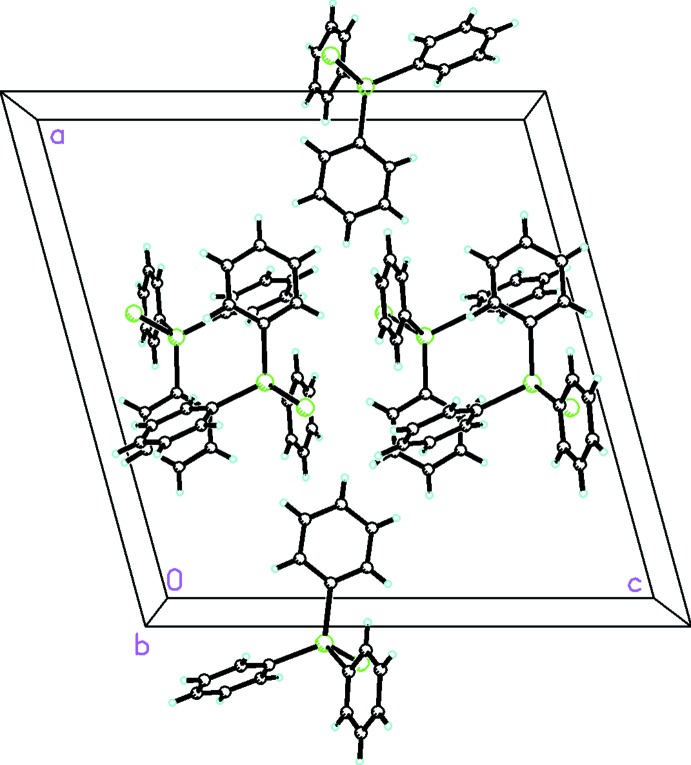
Packing diagram of C18H15ClTe viewed along b axis.

## supplementary crystallographic information

### 1. Introduction

In recent years organotellurium compounds have been widely used as ligands forming complexes with supra­molecular behaviour (Santos *et al.*, 2007; Teikink *et al.*, 2010; Srivastava *et al.*, 2011). These compounds have inter­esting applications as anti­leishmanial and anti­bacterial agents (Lima *et al.*, 2009; Soni *et al.*, 2005). The crystal structures of related compounds, tris­(penta­fluoro­phenyl)­tellurium chloride, (C_6_H_5_)_3_TeCl and tris­(penta­fluoro­phenyl)­tellurium bromide, (C_6_H_5_)_3_TeBr have been reported earlier (Klapötke *et al.*, 2001; Naumann *et al.*, 2002). As part of our investigations on the chalcogen bearing compounds (Srivastava *et al.* 2010; Rastogi *et al.* 2011), we herein report the synthesis and X-ray crystal structure analysis of the title compound, tri­phenyl­tellurium chloride.

### 2.1. Synthesis and crystallization

The title compound was prepared by the modified procedure described earlier (Günther *et al.*,1974). A mixture of TeCl_4_ (26.8 g, 0.1 mol) and AlCl_3_ (39.9 g, 0.3 mol) in 300 mL dry benzene was placed into a 500 mL two-necked, round-bottom flask equipped with a magnetic stirring bar, a nitro­gen inlet and a reflux condenser. The reflux condenser was connected with Tygon tubing to a gas dispersion tube immersed in water containing phenolphthalein indicator. The reaction mixture was heated to reflux under nitro­gen. Vigorous hydrogen chloride evolution occurred immediately. The hydrogen chloride was swept through the condenser into phenolphthalein solution by nitro­gen and titrated with NaOH solution. The reaction mixture was poured into 400 mL of ice and water, when three equivalents of HCl had evolved. A dark colored solid was separated by filtration of the quenched reaction mixture and dissolved in minimum amount of boiling water. The hot mixture was then quickly filtered to give a clear colorless solution. On cooling the filtrate, a white crystalline solid of tri­phenyl­tellurium chloride separated out. The compound was crystallized in ethanol and chloro­form mixture (60:40) to give white crystals suitable for X-ray analysis in 72% yield. M.P. 249-250 °C. Anal. calc. for C_18_H_15_ClTe(%): C,54.82; H,3.83; Cl,8.99; Te,32.35. Found: C,54.88; H,3.86; Cl,9.16; Te,32.30.

### 2.2. Refinement

H atoms were positioned geometrically and refined using the riding model, with C–H distance of 0.95 Å, with *U_iso_* (H) = 1.20 *U_eq_* (C) atoms.

### 3. Results and discussion

The molecular structure of the title compound, C18H15ClTe, is shown in Fig.1. The asymmetric unit of the structure contains two molecules which are in inverted orientations. The molecule displays a tetra­hedral geometry around the Te atom (sum of bond angles, 436.56°) in spite of being five electron domains. This attributes the fact that the lone pair is not sterically active. This is in contrast with the reported structure of distorted o­cta­hedral geometry for (C_6_F_5_)_3_TeCl (Klapotke *et al.*, 2001) and trigonal bipyramidal geometry for (C_6_F_5_)_3_TeBr (Naumann *et al.*, 2002). This clearly indicates that there is no effect of free electron pair at Te in the present structure. The dihedral angles between the mean planes of the three phenyl rings C7A–C12A, C1A–C6A, C13A–C18A in molecule A and C7B–C12B, C1B–C6B, C13B–C18B in molecule B are 76.51 (16)/73.75 (16) and 78.60 (17)/77.67 (16)°, respectively, in the two molecules indicating that there is no conjugation between three aromatic rings. The two phenyl rings at C7 and C13 are inclined at an angle of 71.06 (17)° in molecule A and 79.11 (16)° in molecule B. The crystal packing is stabilized by eight C—H···π inter­molecular inter­actions (Table 1, Fig.2).

### Crystal data

**Table:**

C_18_H_15_ClTe	*F*(000) = 1536
*M**_r_* = 394.35	*D*_x_ = 1.635 Mg m^−^^3^
Monoclinic, *P*2_1_/*c*	Cu *K*α radiation, λ = 1.54184 Å
*a* = 18.7514 (3) Å	Cell parameters from 8646 reflections
*b* = 9.60800 (15) Å	θ = 3.0–75.3°
*c* = 18.4367 (3) Å	µ = 16.07 mm^−^^1^
β = 105.2453 (16)°	*T* = 123 K
*V* = 3204.74 (9) Å^3^	Prism, colorless
*Z* = 8	0.25 × 0.12 × 0.08 mm

### Data collection

**Table:**

Agilent Xcalibur (Ruby, Gemini) diffractometer	6446 independent reflections
Radiation source: Enhance(Cu)X-ray Source	5854 reflections with *I* > 2σ(*I*)
Detector resolution: 10.5081 pixels mm^-1^	*R*_int_ = 0.051
ω scans	θ_max_ = 75.5°, θ_min_ = 4.9°
Absorption correction: multi-scan (*CrysAlis PRO*; Agilent, 2012)	*h* = −23→22
*T*_min_ = 0.215, *T*_max_ = 1.000	*k* = −11→11
12435 measured reflections	*l* = −15→22

### Refinement

**Table:**

Refinement on *F*^2^	0 restraints
Least-squares matrix: full	Hydrogen site location: inferred from neighbouring sites
*R*[*F*^2^ > 2σ(*F*^2^)] = 0.034	H-atom parameters constrained
*wR*(*F*^2^) = 0.077	*w* = 1/[σ^2^(*F*_o_^2^) + (0.0353*P*)^2^] where *P* = (*F*_o_^2^ + 2*F*_c_^2^)/3
*S* = 1.04	(Δ/σ)_max_ = 0.002
6446 reflections	Δρ_max_ = 0.98 e Å^−^^3^
361 parameters	Δρ_min_ = −1.24 e Å^−^^3^

### Special details

**Table:**

Geometry. All e.s.d.'s (except the e.s.d. in the dihedral angle between two l.s. planes) are estimated using the full covariance matrix. The cell e.s.d.'s are taken into account individually in the estimation of e.s.d.'s in distances, angles and torsion angles; correlations between e.s.d.'s in cell parameters are only used when they are defined by crystal symmetry. An approximate (isotropic) treatment of cell e.s.d.'s is used for estimating e.s.d.'s involving l.s. planes.

### Fractional atomic coordinates and isotropic or equivalent isotropic displacement parameters (Å^2^)

**Table:**

	*x*	*y*	*z*	*U*_iso_*/*U*_eq_	
Te1	0.94906 (2)	0.68564 (2)	0.31239 (2)	0.01281 (7)	
Cl1A	0.89902 (5)	0.50007 (10)	0.36822 (5)	0.02617 (18)	
C1A	0.90760 (17)	0.8720 (3)	0.34765 (15)	0.0117 (5)	
C2A	0.95451 (18)	0.9705 (4)	0.39152 (17)	0.0174 (6)	
H2AA	1.0060	0.9520	0.4089	0.021*	
C3A	0.9264 (2)	1.0956 (4)	0.40997 (19)	0.0221 (7)	
H3AA	0.9586	1.1625	0.4395	0.027*	
C4A	0.8510 (2)	1.1227 (4)	0.38521 (19)	0.0203 (7)	
H4AA	0.8318	1.2084	0.3976	0.024*	
C5A	0.80402 (18)	1.0247 (4)	0.34240 (17)	0.0177 (6)	
H5AA	0.7525	1.0427	0.3260	0.021*	
C6A	0.83215 (17)	0.9001 (4)	0.32338 (16)	0.0136 (6)	
H6AA	0.7997	0.8337	0.2936	0.016*	
C7A	0.90531 (16)	0.6542 (4)	0.19509 (16)	0.0132 (6)	
C8A	0.9106 (2)	0.7593 (4)	0.14490 (18)	0.0192 (6)	
H8AA	0.9337	0.8449	0.1632	0.023*	
C9A	0.8819 (2)	0.7394 (4)	0.06757 (18)	0.0241 (7)	
H9AA	0.8860	0.8112	0.0334	0.029*	
C10A	0.8474 (2)	0.6147 (4)	0.04067 (18)	0.0253 (8)	
H10A	0.8277	0.6015	−0.0119	0.030*	
C11A	0.84169 (19)	0.5095 (4)	0.0903 (2)	0.0217 (7)	
H11A	0.8182	0.4243	0.0717	0.026*	
C12A	0.87045 (18)	0.5289 (4)	0.16747 (19)	0.0175 (6)	
H12A	0.8664	0.4569	0.2014	0.021*	
C13A	1.06448 (16)	0.6564 (3)	0.35559 (16)	0.0113 (5)	
C14A	1.11265 (18)	0.6647 (4)	0.30923 (16)	0.0157 (6)	
H14A	1.0936	0.6821	0.2570	0.019*	
C15A	1.18813 (18)	0.6477 (4)	0.33918 (18)	0.0183 (6)	
H15A	1.2206	0.6547	0.3076	0.022*	
C16A	1.21641 (18)	0.6202 (4)	0.41587 (19)	0.0196 (7)	
H16A	1.2681	0.6082	0.4363	0.023*	
C17A	1.16919 (19)	0.6103 (4)	0.46211 (17)	0.0175 (6)	
H17A	1.1884	0.5911	0.5141	0.021*	
C18A	1.09335 (18)	0.6286 (3)	0.43220 (16)	0.0143 (6)	
H18A	1.0611	0.6220	0.4641	0.017*	
Te2	0.45436 (2)	0.55648 (2)	0.33256 (2)	0.01326 (7)	
Cl1B	0.40664 (5)	0.72841 (10)	0.39883 (5)	0.02692 (18)	
C1B	0.40378 (16)	0.5962 (4)	0.21742 (16)	0.0139 (6)	
C2B	0.4015 (2)	0.4914 (4)	0.16450 (18)	0.0191 (6)	
H2BA	0.4224	0.4030	0.1806	0.023*	
C3B	0.3690 (2)	0.5145 (4)	0.08827 (18)	0.0216 (7)	
H3BA	0.3680	0.4424	0.0528	0.026*	
C4B	0.33825 (19)	0.6434 (4)	0.06456 (18)	0.0207 (7)	
H4BA	0.3159	0.6593	0.0127	0.025*	
C5B	0.33996 (18)	0.7490 (4)	0.11611 (18)	0.0186 (6)	
H5BA	0.3191	0.8373	0.0995	0.022*	
C6B	0.37254 (17)	0.7257 (3)	0.19282 (17)	0.0146 (6)	
H6BA	0.3734	0.7980	0.2282	0.017*	
C7B	0.56983 (16)	0.5920 (3)	0.36148 (16)	0.0110 (5)	
C8B	0.60684 (17)	0.5895 (4)	0.30504 (15)	0.0138 (6)	
H8BA	0.5800	0.5717	0.2546	0.017*	
C9B	0.68260 (19)	0.6128 (4)	0.32186 (17)	0.0182 (6)	
H9BA	0.7073	0.6108	0.2830	0.022*	
C10B	0.72222 (18)	0.6392 (4)	0.39582 (18)	0.0188 (6)	
H10B	0.7740	0.6550	0.4075	0.023*	
C11B	0.68595 (18)	0.6424 (4)	0.45235 (17)	0.0164 (6)	
H11B	0.7130	0.6611	0.5027	0.020*	
C12B	0.60982 (18)	0.6181 (4)	0.43578 (16)	0.0148 (6)	
H12B	0.5853	0.6194	0.4748	0.018*	
C13B	0.41530 (17)	0.3643 (3)	0.36394 (15)	0.0121 (6)	
C14B	0.46223 (18)	0.2532 (4)	0.39384 (17)	0.0173 (6)	
H14B	0.5143	0.2645	0.4044	0.021*	
C15B	0.4328 (2)	0.1266 (4)	0.40814 (19)	0.0229 (7)	
H15B	0.4649	0.0514	0.4281	0.027*	
C16B	0.3565 (2)	0.1092 (4)	0.39335 (18)	0.0219 (7)	
H16B	0.3365	0.0222	0.4027	0.026*	
C17B	0.30978 (19)	0.2199 (4)	0.36487 (18)	0.0205 (7)	
H17B	0.2578	0.2090	0.3557	0.025*	
C18B	0.33869 (17)	0.3461 (4)	0.34975 (16)	0.0145 (6)	
H18B	0.3063	0.4208	0.3296	0.017*	

### Atomic displacement parameters (Å^2^)

**Table:**

	*U*^11^	*U*^22^	*U*^33^	*U*^12^	*U*^13^	*U*^23^
Te1	0.01195 (10)	0.01092 (11)	0.01414 (9)	0.00276 (7)	0.00093 (7)	0.00010 (6)
Cl1A	0.0262 (4)	0.0210 (4)	0.0342 (4)	0.0001 (3)	0.0131 (3)	0.0099 (3)
C1A	0.0140 (14)	0.0102 (14)	0.0114 (11)	0.0027 (11)	0.0043 (10)	−0.0017 (10)
C2A	0.0125 (14)	0.0214 (18)	0.0168 (13)	−0.0011 (13)	0.0010 (11)	−0.0029 (12)
C3A	0.0222 (17)	0.0186 (18)	0.0268 (16)	−0.0052 (14)	0.0085 (13)	−0.0093 (14)
C4A	0.0259 (17)	0.0130 (16)	0.0254 (15)	0.0060 (13)	0.0131 (13)	−0.0020 (12)
C5A	0.0143 (15)	0.0195 (17)	0.0197 (13)	0.0084 (13)	0.0051 (11)	0.0036 (12)
C6A	0.0124 (14)	0.0135 (15)	0.0131 (12)	0.0006 (12)	0.0004 (10)	−0.0001 (11)
C7A	0.0080 (13)	0.0125 (15)	0.0176 (13)	0.0057 (11)	0.0008 (10)	−0.0012 (11)
C8A	0.0234 (17)	0.0118 (16)	0.0191 (14)	0.0002 (13)	0.0001 (12)	−0.0017 (12)
C9A	0.0317 (19)	0.0200 (18)	0.0170 (14)	0.0076 (15)	0.0002 (13)	0.0034 (13)
C10A	0.0245 (17)	0.029 (2)	0.0171 (14)	0.0161 (16)	−0.0045 (12)	−0.0083 (14)
C11A	0.0174 (16)	0.0165 (17)	0.0271 (16)	0.0042 (13)	−0.0014 (12)	−0.0115 (13)
C12A	0.0133 (14)	0.0129 (16)	0.0249 (15)	0.0009 (12)	0.0026 (12)	−0.0050 (12)
C13A	0.0082 (12)	0.0096 (14)	0.0134 (12)	0.0003 (11)	−0.0019 (10)	−0.0023 (10)
C14A	0.0189 (15)	0.0152 (16)	0.0122 (12)	0.0010 (12)	0.0027 (11)	−0.0017 (11)
C15A	0.0134 (14)	0.0190 (17)	0.0240 (15)	0.0018 (13)	0.0071 (12)	−0.0047 (13)
C16A	0.0110 (14)	0.0183 (17)	0.0250 (15)	0.0030 (13)	−0.0030 (12)	−0.0043 (13)
C17A	0.0177 (15)	0.0157 (16)	0.0136 (12)	0.0049 (13)	−0.0057 (11)	−0.0012 (11)
C18A	0.0165 (15)	0.0129 (15)	0.0130 (12)	0.0007 (12)	0.0031 (11)	−0.0015 (11)
Te2	0.01147 (10)	0.01182 (11)	0.01491 (10)	−0.00067 (7)	0.00070 (7)	0.00025 (6)
Cl1B	0.0253 (4)	0.0222 (4)	0.0349 (4)	0.0029 (3)	0.0109 (3)	−0.0098 (3)
C1B	0.0070 (13)	0.0157 (16)	0.0165 (13)	−0.0049 (12)	−0.0012 (10)	−0.0002 (12)
C2B	0.0233 (17)	0.0115 (16)	0.0204 (14)	−0.0034 (13)	0.0020 (12)	0.0003 (12)
C3B	0.0301 (18)	0.0161 (17)	0.0172 (14)	−0.0052 (14)	0.0038 (13)	−0.0037 (12)
C4B	0.0191 (16)	0.0219 (18)	0.0175 (14)	−0.0076 (14)	−0.0019 (12)	0.0066 (13)
C5B	0.0135 (15)	0.0165 (17)	0.0224 (15)	−0.0028 (12)	−0.0012 (12)	0.0078 (13)
C6B	0.0111 (13)	0.0098 (15)	0.0203 (14)	−0.0022 (12)	−0.0004 (11)	0.0013 (11)
C7B	0.0076 (12)	0.0088 (14)	0.0144 (12)	0.0018 (11)	−0.0011 (10)	0.0018 (10)
C8B	0.0166 (15)	0.0140 (15)	0.0091 (11)	−0.0023 (12)	0.0003 (10)	0.0027 (10)
C9B	0.0186 (15)	0.0202 (18)	0.0171 (14)	−0.0020 (13)	0.0070 (11)	0.0059 (12)
C10B	0.0139 (14)	0.0168 (17)	0.0228 (15)	−0.0017 (13)	−0.0002 (12)	0.0007 (12)
C11B	0.0138 (14)	0.0162 (16)	0.0157 (13)	−0.0014 (12)	−0.0024 (11)	−0.0029 (11)
C12B	0.0166 (15)	0.0159 (16)	0.0119 (12)	0.0028 (12)	0.0035 (11)	−0.0021 (11)
C13B	0.0152 (14)	0.0121 (15)	0.0081 (11)	−0.0024 (12)	0.0016 (10)	0.0029 (10)
C14B	0.0152 (15)	0.0185 (17)	0.0187 (13)	0.0029 (13)	0.0052 (11)	0.0033 (12)
C15B	0.0334 (19)	0.0160 (17)	0.0208 (14)	0.0064 (15)	0.0100 (13)	0.0056 (12)
C16B	0.0321 (19)	0.0182 (17)	0.0181 (14)	−0.0100 (14)	0.0116 (13)	−0.0001 (12)
C17B	0.0178 (16)	0.0253 (19)	0.0187 (13)	−0.0090 (14)	0.0056 (12)	−0.0025 (13)
C18B	0.0116 (14)	0.0165 (16)	0.0143 (12)	0.0000 (12)	0.0017 (10)	−0.0006 (11)

### Geometric parameters (Å, º)

**Table:**

Te1—C13A	2.119 (3)	Te2—C7B	2.117 (3)
Te1—C1A	2.121 (3)	Te2—C1B	2.120 (3)
Te1—C7A	2.123 (3)	Te2—C13B	2.122 (3)
Te1—Cl1A	2.3720 (9)	Te2—Cl1B	2.3659 (9)
C1A—C6A	1.394 (4)	C1B—C2B	1.395 (5)
C1A—C2A	1.396 (4)	C1B—C6B	1.399 (5)
C2A—C3A	1.390 (5)	C2B—C3B	1.395 (5)
C2A—H2AA	0.9500	C2B—H2BA	0.9500
C3A—C4A	1.390 (5)	C3B—C4B	1.387 (5)
C3A—H3AA	0.9500	C3B—H3BA	0.9500
C4A—C5A	1.386 (5)	C4B—C5B	1.385 (5)
C4A—H4AA	0.9500	C4B—H4BA	0.9500
C5A—C6A	1.390 (5)	C5B—C6B	1.403 (4)
C5A—H5AA	0.9500	C5B—H5BA	0.9500
C6A—H6AA	0.9500	C6B—H6BA	0.9500
C7A—C8A	1.390 (5)	C7B—C8B	1.395 (4)
C7A—C12A	1.400 (5)	C7B—C12B	1.401 (4)
C8A—C9A	1.398 (4)	C8B—C9B	1.390 (5)
C8A—H8AA	0.9500	C8B—H8BA	0.9500
C9A—C10A	1.390 (6)	C9B—C10B	1.394 (5)
C9A—H9AA	0.9500	C9B—H9BA	0.9500
C10A—C11A	1.386 (6)	C10B—C11B	1.387 (5)
C10A—H10A	0.9500	C10B—H10B	0.9500
C11A—C12A	1.396 (5)	C11B—C12B	1.398 (5)
C11A—H11A	0.9500	C11B—H11B	0.9500
C12A—H12A	0.9500	C12B—H12B	0.9500
C13A—C18A	1.399 (4)	C13B—C14B	1.401 (5)
C13A—C14A	1.400 (4)	C13B—C18B	1.402 (4)
C14A—C15A	1.387 (5)	C14B—C15B	1.389 (5)
C14A—H14A	0.9500	C14B—H14B	0.9500
C15A—C16A	1.399 (5)	C15B—C16B	1.395 (6)
C15A—H15A	0.9500	C15B—H15B	0.9500
C16A—C17A	1.384 (5)	C16B—C17B	1.390 (6)
C16A—H16A	0.9500	C16B—H16B	0.9500
C17A—C18A	1.395 (5)	C17B—C18B	1.386 (5)
C17A—H17A	0.9500	C17B—H17B	0.9500
C18A—H18A	0.9500	C18B—H18B	0.9500
			
C13A—Te1—C1A	114.64 (12)	C7B—Te2—C1B	112.45 (11)
C13A—Te1—C7A	116.54 (11)	C7B—Te2—C13B	118.37 (12)
C1A—Te1—C7A	110.95 (11)	C1B—Te2—C13B	109.51 (11)
C13A—Te1—Cl1A	102.64 (9)	C7B—Te2—Cl1B	104.96 (9)
C1A—Te1—Cl1A	106.43 (9)	C1B—Te2—Cl1B	105.13 (10)
C7A—Te1—Cl1A	104.14 (10)	C13B—Te2—Cl1B	105.20 (9)
C6A—C1A—C2A	119.1 (3)	C2B—C1B—C6B	118.8 (3)
C6A—C1A—Te1	119.2 (2)	C2B—C1B—Te2	119.6 (2)
C2A—C1A—Te1	121.6 (2)	C6B—C1B—Te2	121.6 (2)
C3A—C2A—C1A	120.4 (3)	C1B—C2B—C3B	121.0 (3)
C3A—C2A—H2AA	119.8	C1B—C2B—H2BA	119.5
C1A—C2A—H2AA	119.8	C3B—C2B—H2BA	119.5
C2A—C3A—C4A	120.0 (3)	C4B—C3B—C2B	119.7 (3)
C2A—C3A—H3AA	120.0	C4B—C3B—H3BA	120.2
C4A—C3A—H3AA	120.0	C2B—C3B—H3BA	120.2
C5A—C4A—C3A	119.9 (3)	C5B—C4B—C3B	120.3 (3)
C5A—C4A—H4AA	120.0	C5B—C4B—H4BA	119.8
C3A—C4A—H4AA	120.0	C3B—C4B—H4BA	119.8
C4A—C5A—C6A	120.1 (3)	C4B—C5B—C6B	120.0 (3)
C4A—C5A—H5AA	119.9	C4B—C5B—H5BA	120.0
C6A—C5A—H5AA	119.9	C6B—C5B—H5BA	120.0
C5A—C6A—C1A	120.4 (3)	C1B—C6B—C5B	120.2 (3)
C5A—C6A—H6AA	119.8	C1B—C6B—H6BA	119.9
C1A—C6A—H6AA	119.8	C5B—C6B—H6BA	119.9
C8A—C7A—C12A	119.3 (3)	C8B—C7B—C12B	119.3 (3)
C8A—C7A—Te1	119.9 (2)	C8B—C7B—Te2	119.1 (2)
C12A—C7A—Te1	120.7 (2)	C12B—C7B—Te2	121.6 (2)
C7A—C8A—C9A	120.3 (3)	C9B—C8B—C7B	120.7 (3)
C7A—C8A—H8AA	119.9	C9B—C8B—H8BA	119.6
C9A—C8A—H8AA	119.9	C7B—C8B—H8BA	119.6
C10A—C9A—C8A	120.0 (3)	C8B—C9B—C10B	119.9 (3)
C10A—C9A—H9AA	120.0	C8B—C9B—H9BA	120.1
C8A—C9A—H9AA	120.0	C10B—C9B—H9BA	120.1
C11A—C10A—C9A	120.2 (3)	C11B—C10B—C9B	119.9 (3)
C11A—C10A—H10A	119.9	C11B—C10B—H10B	120.0
C9A—C10A—H10A	119.9	C9B—C10B—H10B	120.0
C10A—C11A—C12A	119.9 (3)	C10B—C11B—C12B	120.4 (3)
C10A—C11A—H11A	120.0	C10B—C11B—H11B	119.8
C12A—C11A—H11A	120.0	C12B—C11B—H11B	119.8
C11A—C12A—C7A	120.3 (3)	C11B—C12B—C7B	119.8 (3)
C11A—C12A—H12A	119.9	C11B—C12B—H12B	120.1
C7A—C12A—H12A	119.9	C7B—C12B—H12B	120.1
C18A—C13A—C14A	119.1 (3)	C14B—C13B—C18B	119.0 (3)
C18A—C13A—Te1	119.4 (2)	C14B—C13B—Te2	123.0 (2)
C14A—C13A—Te1	121.5 (2)	C18B—C13B—Te2	117.9 (2)
C15A—C14A—C13A	120.3 (3)	C15B—C14B—C13B	120.2 (3)
C15A—C14A—H14A	119.8	C15B—C14B—H14B	119.9
C13A—C14A—H14A	119.8	C13B—C14B—H14B	119.9
C14A—C15A—C16A	120.1 (3)	C14B—C15B—C16B	120.4 (3)
C14A—C15A—H15A	120.0	C14B—C15B—H15B	119.8
C16A—C15A—H15A	120.0	C16B—C15B—H15B	119.8
C17A—C16A—C15A	120.1 (3)	C17B—C16B—C15B	119.6 (3)
C17A—C16A—H16A	119.9	C17B—C16B—H16B	120.2
C15A—C16A—H16A	119.9	C15B—C16B—H16B	120.2
C16A—C17A—C18A	119.9 (3)	C18B—C17B—C16B	120.3 (3)
C16A—C17A—H17A	120.0	C18B—C17B—H17B	119.9
C18A—C17A—H17A	120.0	C16B—C17B—H17B	119.9
C17A—C18A—C13A	120.5 (3)	C17B—C18B—C13B	120.5 (3)
C17A—C18A—H18A	119.8	C17B—C18B—H18B	119.7
C13A—C18A—H18A	119.8	C13B—C18B—H18B	119.7
			
C6A—C1A—C2A—C3A	−0.7 (5)	C6B—C1B—C2B—C3B	0.2 (5)
Te1—C1A—C2A—C3A	176.0 (3)	Te2—C1B—C2B—C3B	−179.6 (3)
C1A—C2A—C3A—C4A	0.5 (5)	C1B—C2B—C3B—C4B	−0.2 (6)
C2A—C3A—C4A—C5A	0.3 (5)	C2B—C3B—C4B—C5B	0.3 (5)
C3A—C4A—C5A—C6A	−0.8 (5)	C3B—C4B—C5B—C6B	−0.4 (5)
C4A—C5A—C6A—C1A	0.6 (5)	C2B—C1B—C6B—C5B	−0.3 (5)
C2A—C1A—C6A—C5A	0.2 (5)	Te2—C1B—C6B—C5B	179.5 (2)
Te1—C1A—C6A—C5A	−176.6 (2)	C4B—C5B—C6B—C1B	0.4 (5)
C12A—C7A—C8A—C9A	−0.6 (5)	C12B—C7B—C8B—C9B	−0.1 (5)
Te1—C7A—C8A—C9A	180.0 (3)	Te2—C7B—C8B—C9B	179.8 (3)
C7A—C8A—C9A—C10A	0.6 (6)	C7B—C8B—C9B—C10B	−0.1 (5)
C8A—C9A—C10A—C11A	−0.4 (6)	C8B—C9B—C10B—C11B	−0.1 (6)
C9A—C10A—C11A—C12A	0.2 (5)	C9B—C10B—C11B—C12B	0.5 (6)
C10A—C11A—C12A—C7A	−0.2 (5)	C10B—C11B—C12B—C7B	−0.7 (5)
C8A—C7A—C12A—C11A	0.4 (5)	C8B—C7B—C12B—C11B	0.5 (5)
Te1—C7A—C12A—C11A	179.8 (2)	Te2—C7B—C12B—C11B	−179.4 (3)
C18A—C13A—C14A—C15A	−1.0 (5)	C18B—C13B—C14B—C15B	0.8 (4)
Te1—C13A—C14A—C15A	178.8 (3)	Te2—C13B—C14B—C15B	−175.3 (2)
C13A—C14A—C15A—C16A	0.9 (5)	C13B—C14B—C15B—C16B	−0.4 (5)
C14A—C15A—C16A—C17A	−0.2 (6)	C14B—C15B—C16B—C17B	−0.7 (5)
C15A—C16A—C17A—C18A	−0.3 (6)	C15B—C16B—C17B—C18B	1.3 (5)
C16A—C17A—C18A—C13A	0.2 (5)	C16B—C17B—C18B—C13B	−0.9 (5)
C14A—C13A—C18A—C17A	0.5 (5)	C14B—C13B—C18B—C17B	−0.2 (4)
Te1—C13A—C18A—C17A	−179.4 (3)	Te2—C13B—C18B—C17B	176.1 (2)

### Hydrogen-bond geometry (Å, º)

Cg1, Cg2, Cg3, Cg4, Cg5 and Cg6 are the centroids of the C1A–C6A, C7A–C12A, C13A–C18A, C1B–C6B, C7B–C12B and C13B–C18B phenyl rings, respectively.

**Table:**

*D*—H···*A*	*D*—H	H···*A*	*D*···*A*	*D*—H···*A*
C2*A*—H2*AA*···*Cg*2^i^	0.95	2.96	3.587 (4)	125
C5*A*—H5*AA*···*Cg*4	0.95	2.65	3.497 (4)	149
C10*A*—H10*A*···*Cg*1^ii^	0.95	2.83	3.580 (4)	137
C5*B*—H5*BA*···*Cg*5^iii^	0.95	2.76	3.532 (4)	139
C11*A*—H11*A*···*Cg*3^iv^	0.95	2.91	3.601 (4)	131
C12*B*—H12*B*···*Cg*6^v^	0.95	2.95	3.671 (3)	134
C14*B*—H14*B*···*Cg*4^vi^	0.95	2.86	3.589 (4)	134
C17*B*—H17*B*···*Cg*2	0.95	2.78	3.679 (4)	158

Symmetry codes: (i) −*x*, *y*+1/2, −*z*+1/2; (ii) *x*, −*y*−1/2, *z*−1/2; (iii) −*x*+1, *y*+1/2, −*z*+1/2; (iv) −*x*, *y*−1/2, −*z*+1/2; (v) −*x*+1, −*y*+1, −*z*+1; (vi) −*x*+1, *y*−1/2, −*z*+1/2.
